# New ZnO-Based Glass Ceramic Sensor for H_2_ and NO_2_ Detection

**DOI:** 10.3390/s17112538

**Published:** 2017-11-03

**Authors:** Mohamed Hassan, Ahmed S. Afify, Mohamed Ataalla, Daniel Milanese, Jean-Marc Tulliani

**Affiliations:** 1 High Institute for Engineering & Technology, 21 K Cairo-Belbeis Rd, Al-Obour 11828, Egypt; dr.m.qader@oi.edu.eg; 2 INSTM R.U PoliTO-LINCE Laboratory, Department of Applied Science and Technology, Politecnico di Torino, Corso Duca degli Abruzzi, 24, 10129 Torino, Italy; ahmed.afify@polito.it; 3 Faculty of Engineering and Technology, Badr University in Cairo (BUC), Badr 11829, Egypt; mohamed.sobhi@buc.edu.eg; 4 Department of Applied Science and Technology, Politecnico di Torino, 10129 Torino, Italy; daniel.milanese@polito.it

**Keywords:** glass ceramics, ZnO, sensors, electrical properties, NO_2_ monitoring

## Abstract

In this study, a glass ceramic with a nominal composition 58ZnO:4Bi_2_O_3_:4WO_3_:33.3B_2_O_3_ was synthesized by melt quenching technique. A gas sensor was then manufactured using a ZnO sol-gel phase as a permanent binder of the glass–ceramic to an alumina substrate having interdigitated electrodes. The film sensitivity towards humidity, NH_3_, H_2_ and NO_2_ was studied at different temperatures. X-ray diffraction technique (XRD), field emission- scanning electron microscopy (FE-SEM) and differential thermal analysis (DTA) were used to characterize the prepared material. Though the response in the sub-ppm NO_2_ concentration range was not explored, the observed results are comparable with the latest found in the literature.

## 1. Introduction

The demand for rugged and reliable chemical sensors able to operate in harsh industrial environments, as well as for public health and security is still high. These sensors have to cover a wide range of industries such as metallurgy, glass, ceramic, paper, automotive, aerospace and energy [1]. To fulfill these requests, emission monitoring sensors able to detect CO, CO_2_, NO_x_ (NO and NO_2_), O_2_, hydrocarbons (HCs) and volatile organic compounds (VOCs) have been developed. Chemical sensors are also used in domestic appliances and air quality monitoring, as well as, for the early detection of smoke/fire and of hazardous chemical agents, to provide safety and security in public places and transportation systems [1]. Yet, despite the high demand, major advances in these sensors in terms of simple structure, lower cost, better selectivity, durability and reliability are always needed. Throughout the years, many materials based on polymers, composites and ceramics have been tested as gas sensors due to their own features and specific operating conditions. However, great attention has been paid to ceramic materials because of their chemical inertness. The base materials most widely investigated for ceramic gas sensors are transition metal oxides based on SnO_2_, TiO_2_, WO_3_, In_2_O_3_, Fe_2_O_3_ and ZnO [2]. ZnO sensing properties have been extensively studied, exhibiting, for example, a high sensitivity to CO, NO, NO_2_, H_2_S, C_2_H_5_OH, NH_3_, CH_4_, SO_2_ gases and acetaldehyde [3,4,5,6]. In addition, many metals such as Al, In, Cu, Fe, Sn, Pt and Ru were proposed as dopants in ZnO gas sensors to improve their sensing properties [3,5]. 

Preparation techniques can considerably affect the physical, chemical and functional properties of semiconducting metal oxide-based gas sensors too. Thus, new synthesis routes, as well as doping, are two promising approaches for the design of highly sensitive and selective gas sensors. Insulated matrix with percolating conductive fillers have been deeply investigated because of the electromechanical interactions between the various phases [7]. Usually, conductor–insulator composites consist of glass, ceramics, or polymer as the insulating phase and of metal, carbon or polymers, as the conducting one [7,8]. In glasses or ceramics as sensing materials, the variation of their electrical properties, such as impedance or capacitance, is exploited for gas detection. There are, however, few papers dealing with glass ceramics as humidity [9] and gas sensors [10,11,12,13,14,15,16]. Thus, based on previous experiences [17,18], in this research, ZnO crystals were grown from a glassy matrix having the composition 58ZnO:4WO_3_:4Bi2O3:33.3B_2_O_3_ by means of a crystallization process. The gas sensing properties of this material were investigated at different operating temperatures with respect to water vapor, NH_3_, H_2_ and NO_2_.

## 2. Materials and Methods

**Figure 1:**
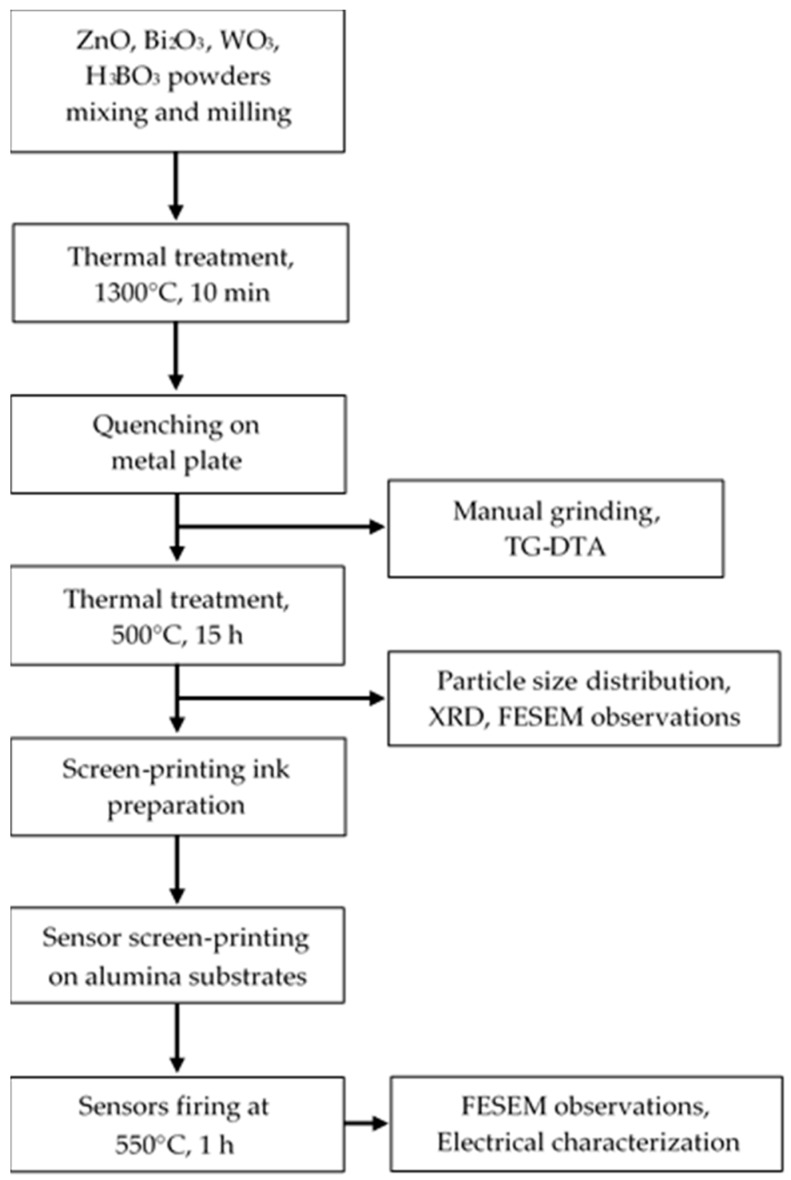
Flow-chart of the sensing material and of the sensors preparation and characterization.

Figure 1 illustrates a flow-chart of the sensing material and of the sensors preparation and characterization.

Powders of zinc oxide (ZnO—Aldrich > 99%), bismuth oxide (Bi_2_O_3_—Alfa Aesar, 99.999% metal basis), tungsten oxide (WO_3_—Aldrich > 99%) and boric acid (H_3_BO_3_—Alfa Aesar, 99.999% metal basis) were used as raw materials for glass preparation; all chemicals were ACS grade.

**Figure 2:**
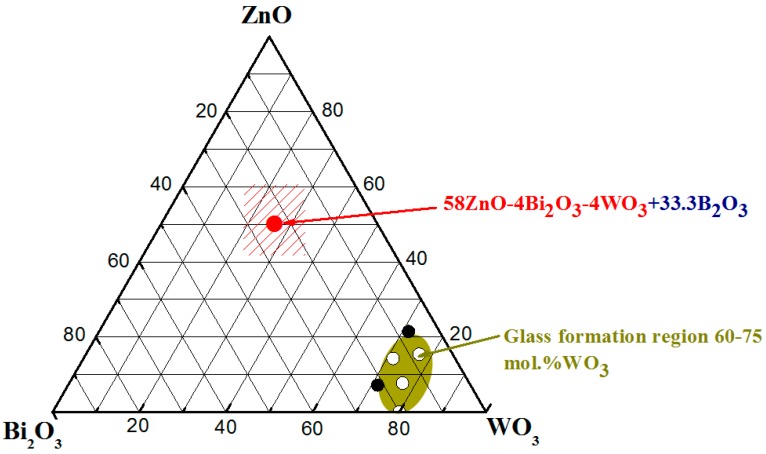
Ternary diagram of ZnO-Bi_2_O_3_-WO_3_ system, where (○) indicate glasses and (●) crystalline phases; (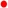) 58ZnO:4Bi_2_O_3_:4WO_3_:33.3B_2_O_3_.

The composition of the studied batch is 58ZnO:4WO_3_:4Bi_2_O_3_:33.3B_2_O_3_ and is reported in the ternary diagram of Figure 2 in mole ratio of oxides. Compositions in mol% and wt% can be calculated by the following Equation (1):

(1) $$x_{A}=\left(X_{A\;}\cdot {\;M}_{A\;}/X_{A\;}\cdot M_{A\;}+X_{B\;}\cdot M_{B\;}\right)\times 100,$$

where: x_A,B_ is the amount in wt% of compound A or B, X_A,B_ is the amount in mol% of compound A or B and M_A,B_ is the molar mass of A or B. ZnO, WO_3_ and Bi_2_O_3_ were used as starting chemicals, while H_3_BO_3_ (boric acid) was used as precursor for the formation of B_2_O_3_ according to the chemical reaction described by Equation (2):

(2) $$2H_{3}BO_{3}\to B_{2}O_{3}+3H_{2}O,$$

After that, the bismuth oxide, boric acid, zinc oxide and tungsten oxide amounts were calculated for 10 g batches. The raw materials were mixed and milled in an agate mortar for 10 min to homogenize the mixture, prior to transfer it into a suitable platinum crucible. Then, the mixture was heated for 10 min at 1300 °C in a muffle furnace, prior to be poured onto a metal plate for quenching with a cooling rate of 10^2^–10^3^ K/s. Finally, the glass was manually ground by means of an agate mortar and an agate pestle. About 50 mg of the prepared powder was placed into a platinum crucible for simultaneous thermogravimetric–differential thermal analysis (TG-DTA, Neztsch STA 409, Selb, Germany) with a reference sample made of alumina powder. The analysis was performed under static air with a 10 °C/min heating rate up to 800 °C. A thermal treatment at 500 °C for 15 h (i.e., at a temperature close to the estimated crystallization temperature onset of glass, T_x_) was then carried out to crystallize ZnO nanoparticles.

Particle size distribution of the powder heat-treated at 500 °C for 15 h was evaluated by laser granulometry (Malvern 3600D, Worcestershire, UK). Laser granulometry measurements were carried out after dispersion in ethanol, before and sonication for 10 min.

X-ray diffraction patterns were collected on prepared powders by means of a X’Pert Powder Pan Analytical Diffractometer, equipped with a Cu anticathode (λ Cu Kα anticathode = 0.154056 nm). Samples were scanned at a rate of 0.02 °/s in the range from 5° to 70° in 2θ. Finally, samples were chromium sputtered for Field Emission Scanning Electron Microscopy (FESEM), Zeiss Merlin, Oberkochen, Germany) observations.

**Figure 3:**
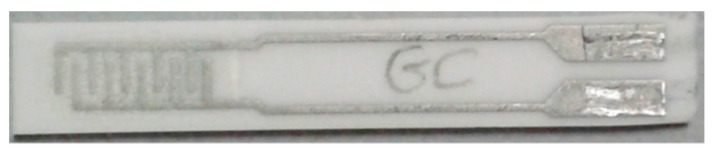
Screen-printed glass ceramic (GC) film onto alumina substrate with Pt interdigitated electrodes.

Glass ceramic (GC) sensors were prepared by adding 2-propanediol to GC powder and mixing in a mortar till getting an acceptable viscous paste. The ink was then manually screen-printed over an alumina substrate onto interdigitated Pt electrodes (Figure 3). These samples were fired at 550 °C for 1 h with a 2 °C/min heating rate.

The sensors were first tested in a laboratory apparatus made of a thermostated chamber, operating at room temperature (RT), in which relative humidity (RH) was varied between 0 and 96% by steps, each one of 3 min [19]. In this RH system, compressed air was separated into two fluxes: one was dehydrated over a chromatography alumina bed, while the second one was directed through two water bubblers (three bubblers if measurements were performed under NH_3_ atmosphere [20]), generating, respectively, a dry and a humid flow [19]. During measurements, the overall airflow (dry + humid one) was kept constant (0.05 L/s). As water is a polarizable molecule and to avoid electrolysis due to the applied voltage, each tested sensor was alimented by an external alternating voltage (V = 3.6 V @ 1 kHz) and constituted a variable resistance of this electrical circuit. A multimeter (Keithley 2700, Beaverton, OR, USA) was used to measure the voltage at the output of the circuit [19]. The sensor resistance was determined by means of a calibrating curve drawn by substituting the sensor, in the circuit, by known resistances and by measuring the voltage across them. RH values were measured by means of a commercial humidity and temperature probe (Delta Ohm DO9406, Padova, Italy). 

For ammonia measurements, the ammonia flow was obtained by diluting an ammonium hydroxide solution (Fluka, Minneapolis, MN, USA) in deionized water (ratio 1:20) into a third drechsel through which the airflow was bubbled [20]. The corresponding ppm of NH_3_ concentration was then estimated by a commercial ammonia probe (Gas Microalert 5, BW Technologies, Calgary, AB, Canada). 

Finally, glass ceramic sensors were tested, first under an oxidizing (NO_2_, 1, 2.5, 5 ppm) and then, under a reducing gas (H_2_, 20, 50, 100 ppm) at a constant flow rate of 200 sccm (standard cubic centimeters). The sensors were investigated in dry air and in humid conditions (50 RH%) and were exposed to each gas concentration for 10 min, while air was flowed for 30 min before increasing the concentration of the targeted gas. Finally, air was flowed during 60 min when changing gas (from NO_2_ to H_2_) and when performing measurements from dry air to humid one. All measurements were carried out using a flow through cell made of Teflon [21]. The sensors were equipped with a screen-printed heater which was alimented by a variable DC power supply to reach the different operating temperatures (from 150 °C to 250 °C). The heater was previously calibrated by means of a pyrometer prior to the sensors’ measurements [21]. The resistances of the sensors were continuously measured with a computer-controlled system by a digital multimeter (Keithley DMM 199, Beaverton, OR, USA) [21].

The sensor response (SR), was defined as the relative variation of the starting resistance in the absence of the test gas, R_0_, and the resistance measured under gas exposure, R_g_, as described by Equation (3):

(3) $$SR={\frac{R_{g}}{R_{0}}}_{},$$

## 3. Results

### 3.1. Differential Thermal Analysis

**Figure 4:**
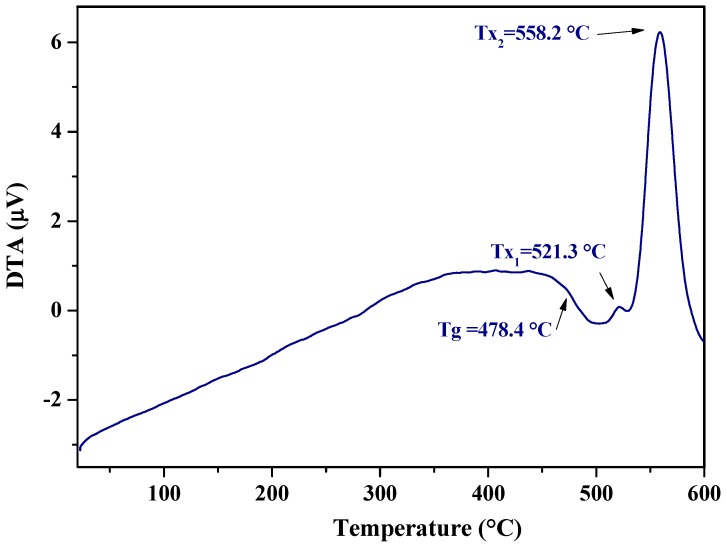
DTA curve of glass with nominal composition 58ZnO:4Bi_2_O_3_:4WO_3_:33.3B_2_O_3_.

The DTA curve of the glass having the composition 58ZnO:4Bi_2_O_3_:4WO_3_:33.3B_2_O_3_ is reported in Figure 4: a glass transition temperature (Tg) is visible at about 478 °C and glass crystallization temperatures (T_x1_ and T_x2_) are evidenced above 500 °C. Considering the estimated crystallization temperature onset of glass T_x_ at 538 °C, the studied glass sample was submitted to a heat treatment for 15 h at the temperature near to the established T_x_ (500 °C).

### 3.2. Particle Size Distribution

**Figure 5:**
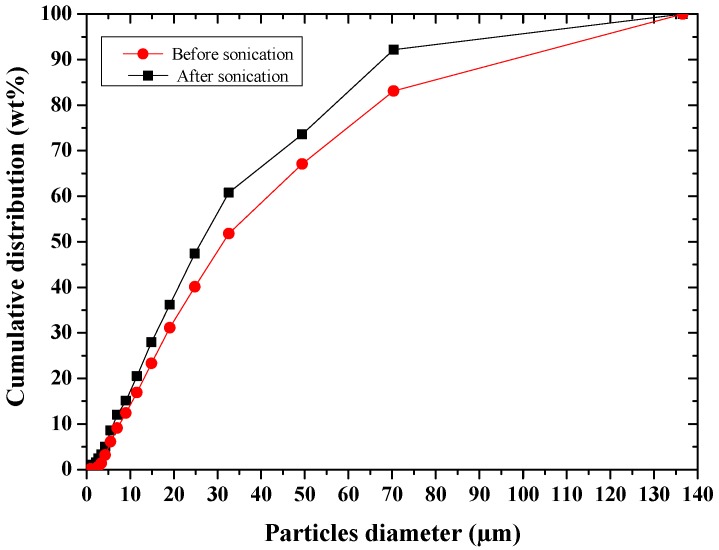
Particle size distribution of glass–ceramic powder before and after sonication.

After sonication of the powder in ethanol for 10 min, Table 1 and Figure 5 showed a slight agglomeration of the particles. The easy deagglomeration of the powder grains suggest that the agglomerates were soft ones.

### 3.3. X-ray Diffraction

**Figure 6:**
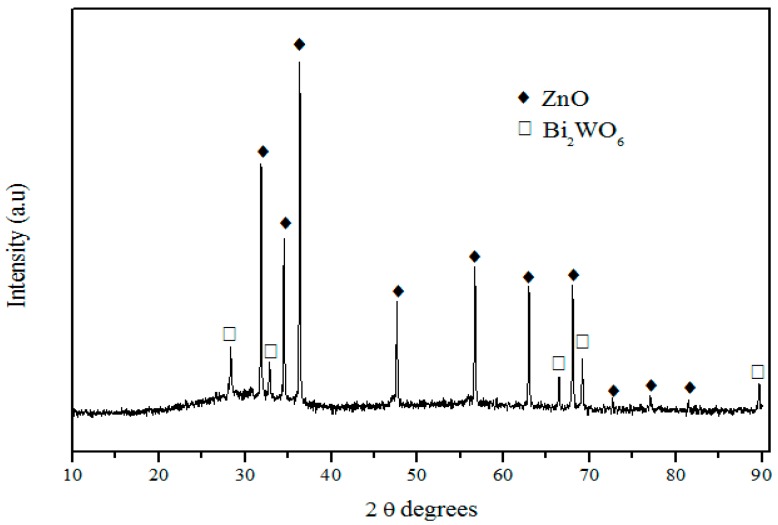
XRD pattern of glass ceramic with nominal composition 58ZnO:4WO_3_:4Bi_2_O_3_:33.4B_2_O_3_ heat treated at 500 °C for 15 h.

XRD pattern shown in Figure 6 evidences the presence of two different crystalline phases: ZnO (JCPDS card no°1389-0511) and Bi_2_WO_6_ (JCPDS card no°1373-2020). ZnO is the main crystalline phase, whereas Bi_2_WO_6_ appears as the secondary crystalline phase. 

### 3.4. SEM/FE-SEM Observations and Elemental Microanalysis

**Figure 7:**
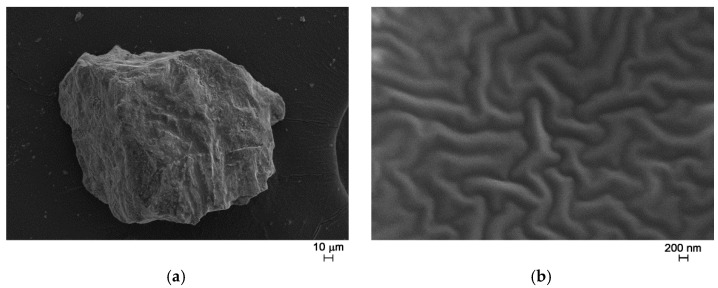
FE-SEM micrographs of as prepared glass ceramic, magnification = 1000× (**a**); 50,000× (**b**).

After the thermal treatment at 500 °C for 15 h, FE-SEM observations showed the presence of a glassy matrix where particles with different size and shape are embedded (Figure 7). 

Elemental microanalysis (performed by means of an Energy Dispersive X-ray Spectrometer, EDS) was performed on different points along the crystals presented in Figure 7 and the matrix. The results confirmed the presence of Zn, W, and Bi atoms. In addition, they revealed a slightly higher percentage of zinc in the crystals than in the glassy matrix (Table 2). 

### 3.5. Sensitivity towards Humidity at Room Temperature

**Figure 8:**
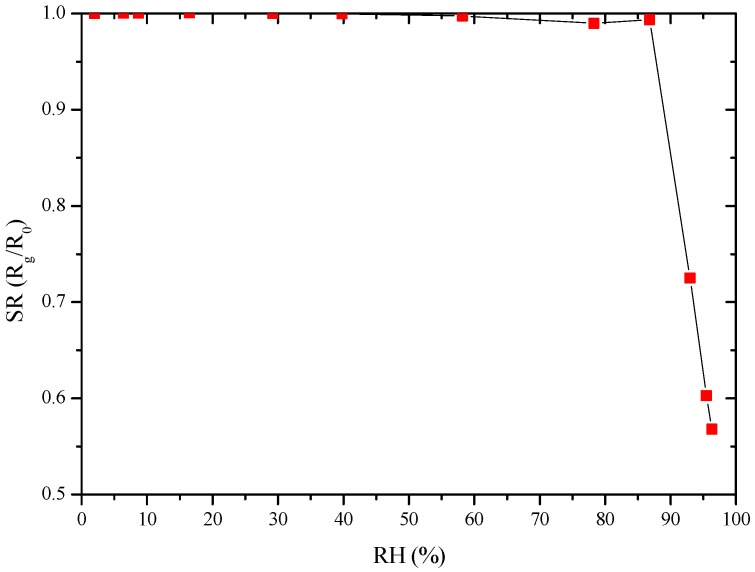
GC sensor response towards RH at RT.

The prepared sensors gave a poor response to water vapor at room temperature up to 87 RH%, as illustrated in Figure 8. This result is encouraging as water vapor is an interfering gas in pollutants detection.

### 3.6. Sensitivity towards NH_3_ at Room Temperature

The sensors gave almost no response towards ammonia at RT in the range 0–75 ppm (curve not shown here). This result is also encouraging as the sensor seems to be selective, at least with respect to ammonia in water.

### 3.7. Sensitivity towards H_2_ and NO_2_ at High Temperature

The screen-printed sensors were then tested by DC resistance measurements under different concentrations of an oxidizing gas (NO_2_ 1, 2.5, 5 ppm), first and then, of a reducing gas (H_2_ 20, 50, 100 ppm), in dry and humid air (50%) at different temperatures (Figure 10). 

**Figure 9:**
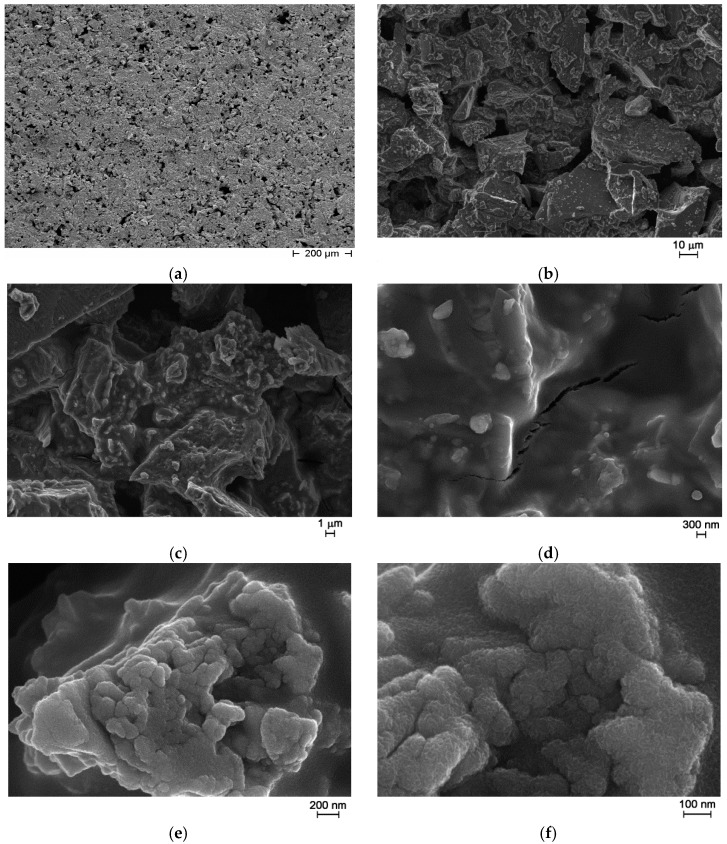
FE-SEM micrographs of sol-gel glass ceramic thick-film: pores in the screen-printed film (**a**–**c**); cracks (**d**); small particles due to sol-gel phase (**e**); ZnO crystals from sol-gel phase (**f**).

From preliminary measurements, no response to the targeted gases was observed and the sensors showed very high resistance values, around 100 GΩ, probably because of the limited adhesion to the alumina substrates. Thus, to improve the adhesion and to decrease the resistance of the screen-printed sensing film, a sol-gel phase based on ZnO was added to the as prepared glass ceramic powder, as follows: 200 mg of zinc acetate dihydrate (Sigma Aldrich, Milan, Italy) was dissolved in 0.625 cm^3^ ethanol, and subsequently 0.066 mL monoethanolamine (MEA, Sigma Aldrich, Milan, Italy) was added under stirring. After 30 min, 0.625 mL of Emflow (a mix of terpinols from Emca Remex, Montgomeryville, PA, USA) were added and after 5 more min of stirring, 70 wt% of liquid was added to the as prepared glass ceramic powder [22]. Then, the paste was screen printed over the alumina substrates with interdigitated Pt electrodes. FE-SEM observations of the new films were done and are reported in Figure 9. After thermal treatment, the screen-printed film is still porous as illustrated in Figure 9a–c. The cracks visible in Figure 9d were already seen on glass ceramics powder alone. The sol-gel phase probably led to the grains visible in Figure 9e, and is made of small particles as illustrated by Figure 9f.

From FE-SEM observations, the thickness of the screen-printed films is around 41 ± 7.2 µm (average of 10 measurements). 

**Figure 10:**
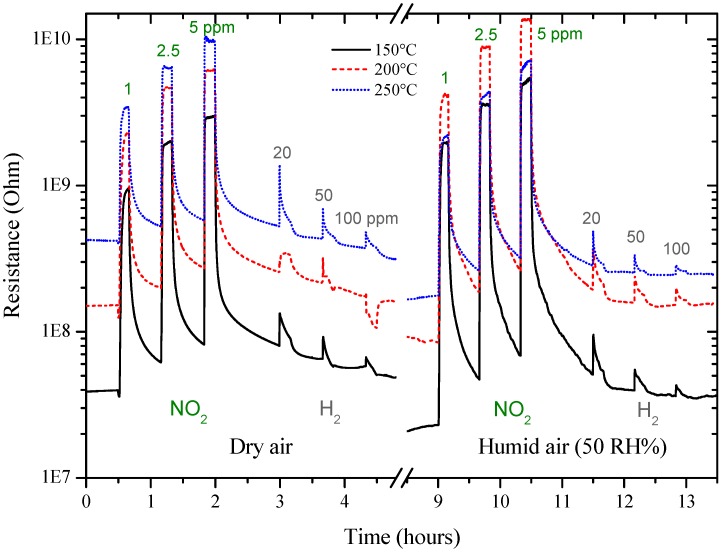
Resistance of sol-gel GC sensor at 150, 200 and 250 °C under NO_2_ and H_2_.

**Figure 11:**
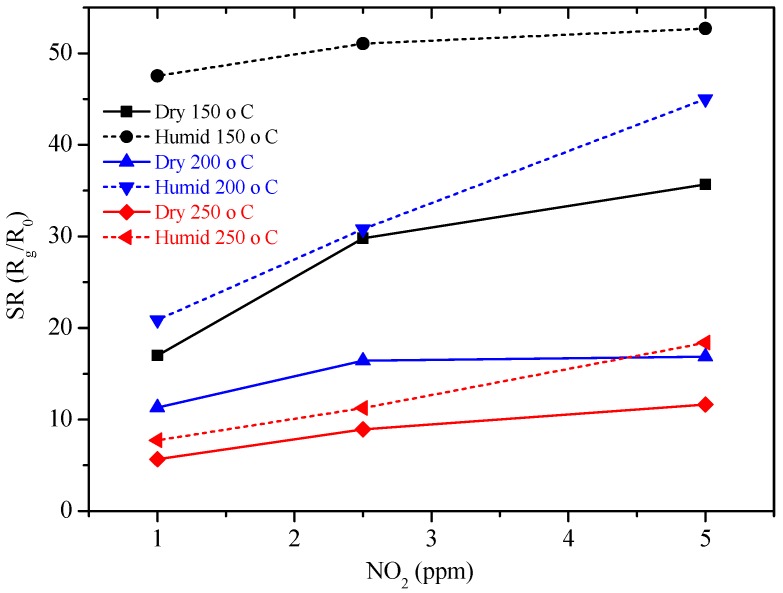
Sensor response of GC sol-gel sensor at 150, 200 and 250 °C under NO_2_ in dry air and under 50 RH%.

**Figure 12:**
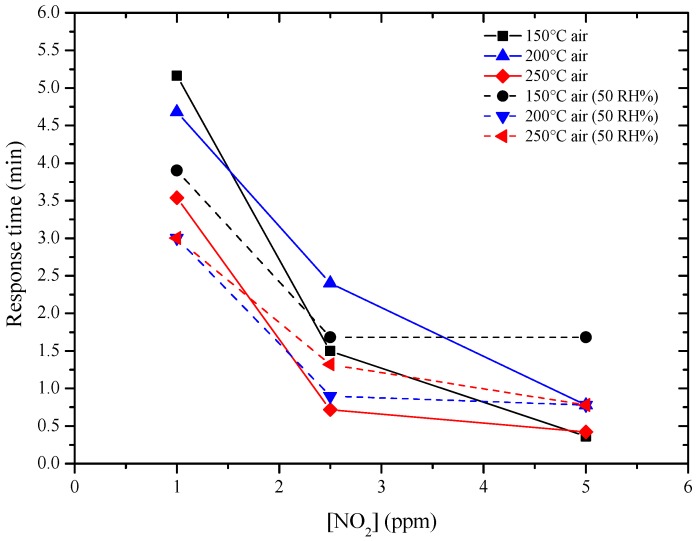
Response time of GC sol-gel sensor in function of NO_2_ concentration and temperature.

The new sensors were then tested by DC resistance measurements with the same pulse program previously used with GC sensors, under the same concentrations of NO_2_ (1, 2.5, 5 ppm) and of H_2_ (20, 50, 100 ppm), in a dry and humid air (50%), at 150, 200 and 250 °C. Sol-gel GC sample seems to be more sensitive to NO_2_ than H_2_, as shown in Figure 10 and the sensor’s resistance always increased, whatever the gas considered. The higher the temperature, the higher the resistance value is (Figure 10). The sensor response to different concentrations of NO_2_ were determined and are shown in Figure 11. Response time, defined as the time required to reach 90% of the final equilibrium resistance value after gas injection, of sol-gel GC sensor towards NO_2_ at 150, 200 and 250 °C were calculated from Figure 10 and are listed in Table 3. Considering these results, the shortest response times towards NO_2_ were reached at 250 °C, under dry and humid air (Figure 12). Recovery times were not determined because during these measurements the sensor resistance was not always allowed to reach the baseline value. Anyway, recovery times are estimated to be always much longer than response times (the resistance value slowly recovers its initial value before gas injection), indicating a rather strong binding between the sensing material and the target gas. 

## 4. Discussion

The sensor response to different concentrations of NO_2_ evidenced that the sensitivity to humidity, almost absent at RT (Figure 8), is enhanced at higher temperatures (150–250 °C, Figure 10 and Figure 11). These results are rather surprising because the mechanisms involved are very different. At high temperatures, chemisorbed water molecules give electrons to the sensing material and, in case of a n-type semiconductor, its resistance decreases. On the contrary, at room temperature, the resistance decrease is due to capillary condensation in pores, or to water molecules hopping, especially at higher RH values. A possible explanation may be because, when present, water molecules form a layer of OH^−^ ions on the oxide surface which tends to lower the number of chemisorbed oxygen species. This surface covering by water molecules reduces the available sites for NO_2_ and H_2_ molecules to adsorb and thus, deteriorates sensor performances [23,24]. In any case, the higher the working temperature, the lower the influence of water vapor is (Figure 11).

Undoped zinc oxide (ZnO) is a well-known intrinsic n-type semiconductor having a wide band gap (3.37 eV) due to the presence of intrinsic defects (oxygen vacancies and zinc interstitials). Bi_2_WO_6_ is also a n-type semiconductor but with a lower band gap (2.7–2.8 eV) respect to ZnO [25]. Then, in the glass ceramic, ZnO and Bi_2_WO_6_ may form a n-n heterojunction if the phases are in contact together. At temperatures between 100 and 500 °C, the presence of atmospheric oxygen leads to the formation of adsorbed layers of molecular (O_2_^−^) and/or atomic (O^−^, O^2−^) oxygen ions. It is known in literature that below 150 °C, the molecular form dominates and above this temperature the ionic species dominate [26]. In any case, these species lead to the formation of a depletion layer at the surface of a semiconducting oxide due to electrons trapping [27,28]. However, in a n-type semiconductor, NO_2_ does not react with pre-adsorbed oxygen and the sensors’ resistance changes are due to a direct chemisorption process [29]. A series of reactions (Equations (4)–(6) leading to nitrates and nitrites formation is reported in Ref. [30]:

(4a) $$NO_{2}+é(c.b.)↔NO_{2}^{-},$$

(4b) $$NO_{2}+V_{O}^{\prime \prime }↔NO_{2}^{-}+V_{O}^{\prime \prime },$$

(5a) $$2NO_{2}+O_{2}^{-}+é(c.b.)↔2NO_{3}^{-},$$

(5b) $$2NO_{2}+O_{2}^{-}+V_{O}^{\prime }(c.b.)↔2NO_{3}^{-}+V_{O}^{\prime },$$

(6) $$NO_{2}+O^{-}↔NO_{3}^{-},$$

In surface reactions (4) and (5), electrons from the conduction band are trapped when surface species are formed, but not in reaction (6). If nitrates are formed according to Equation (6), a further equilibrium must be taken into consideration due to NO_3_^-^ dissociation (Equation (7)):

(7a) $$NO_{3}^{-}+é(c.b.)↔NO+2O^{-},$$

(7b) $$NO_{3}^{-}+V_{O}^{\prime }↔NO+2O_{}^{-}+V_{O}^{\prime \prime },$$

In Figure 10, the resistance of the GC sensor increased upon exposure to NO_2_, as expected for a n-type semiconductor, suggesting that the detection mechanism can be associated with the previous reactions. However, surprisingly, the sensor resistance increased in the presence of H_2_ molecules too (Figure 10). This behavior can be explained considering that n to p transitions have been already observed in several semiconducting oxides throughout the years [31,32]. In the case of ZnO nanotubes, since the surface conduction is due to both electrons and holes contribution, the change of majority carrier density can lead to the inversion of the type of mobile carriers at the surface [32]. Variations of majority carrier density may be explained considering Equations (4a) and (5a) where electrons from the conduction band are trapped by adsorbed H_2_ molecules and then, their concentration becomes lower than the concentration of holes [30,31]. These results could explain the observed n to p transition. To support this hypothesis, this behavior is generally observed in the temperature range 200–250 °C, where the presence of both the molecular and atomic oxygen species is probable [31,32]. However, further investigation is needed to better understand the origin of such anomaly.

Though the response of our sensor in the sub-ppm NO_2_ concentration range was not explored, the observed results are comparable with the latest found in literature for ZnO-based sensors (Table 4).

## 5. Conclusions

A glass–crystalline material containing ZnO as the primary crystalline phase was successfully produced by melt quenching technique. A gas sensor was then fabricated using a ZnO sol-gel phase as a permanent binder of the glass ceramic to the alumina substrate. The sensor response depends on the operating temperature and on the concentration of the target gas. The highest response to NO_2_ was observed at an operating temperature of 150 °C. However, a certain interference with water vapor was evidenced at this temperature, even if the higher the working temperature (250 °C in our case), the lower the influence of water vapor is. Another possible solution to limit water vapor interferences is to reduce the porosity of the film, as already proposed in Ref. [52], although the counterpart can be a certain loss of sensitivity. A last solution could be to grow ZnO nanotubes on top of glass ceramic grains from sol-gel solution [53]. In this case, a higher hydrophobicity of the surface coated with needles can be obtained [53], by mimicking the lotus effect [54]. To conclude, glass ceramic materials are promising in sensors application not only as humidity sensors but also for detecting other gases.

## Figures and Tables

**Table 1:** Diameter in micron of glass ceramic powder before and after sonication.

Cumulative wt% Below	Before Sonication (µm)	After Sonication (µm)
90	97	67
50	32	26
20	14	11

**Table 2:** EDS results of the glass ceramic as prepared material.

At %		
Element	Crystal	Matrix
Zn	84.94	79.94
W	0.85	3.35
Bi	14.21	16.71

**Table 3:** Response time of sol-gel GC sensor towards NO_2_ at 150, 200 and 250 °C.

[gas] (ppm)	1	2.5	5
**Response time (dry air 150 °C)**	5 min 10 s	1 min 30 s	22 s
**Response time (humid air 150 °C)**	3 min 54 s	1 min 40 s	1 min 40 s
**Response time (dry air 200 °C)**	4 min 40 s	2 min 2 s	47 s
**Response time (humid air 200 °C)**	3 min	54 s	47 s
**Response time (dry air 250 °C)**	3 min 32 s	43 s	25 s
**Response time (humid air 250 °C)**	3 min	1 min 19 s	47 s

**Table 4:** Response towards NO_2_ of ZnO-based resistive sensors.

Technological Route	Film Type	NO_2_ Sensor Response (SR)	Conditions of Measurement	Reference
In-situ production of ZnO nanoparticles onto reduced graphene oxide	n.m.^1^	SR = (R_a_ – R_g_)/R_a_ 0.26 under 5 ppm	RT	[33]
Reduced graphene oxide nanosheets-loaded ZnO nanofibers via electrospinning	Thin-film	SR = R_g_/R_a_ ca. 90 under 1 ppm	400 °C	[34]
ZnO nanorods prepared by hydrothermal method	n.m.	SR = R_g_/R_a_ 1.8 under 1 ppm	300 °C	[35]
ZnO/Single Walled Nano-Tubes 1:1 in wt spin coated	Thick-film	SR = R_a_/R_g_ 0.7 under 1 ppm	300 °C	[36]
ZnO nanorods prepared by hydrothermal method	Thin-film	SR = R_g_ − R_a_/R_g_ 12.4 under 0.1 ppm	100 °C	[37]
ZnO produced by wet chemical route	Thin-film	SR = R_a_/R_g_ 1.01 under 2 ppm	300 °C	[38]
ZnO nanoflowers prepared by hydrothermal synthesis + reduced graphene oxide	Thick-film	SR = R_g_/R_a_ ca. 13 under 1 ppm	174 °C	[39]
ZnO nanorods deposited using a wet chemical route	Thin-film	SR = R_g_ − R_a_/R_g_ ca. 5.7 under 20 ppm	175 °C	[40]
Metallic single-walled carbon nanotubes electrodes with ZnO nanowires	Thick-film	SR = R_g_ − R_a_/R_a_ 2 under 2.5 ppm	25 °C	[41]
Soft chemical synthesis of flower-shaped ZnO	Thin-film	SR = R_g_/R_a_ 1.4 under 10 ppm	200 °C	[42]
Electrospun ZnO fibers	Thin-film	SR = R_g_/R_a_ ca. 5.5 under 0.1 ppm	200 °C	[43]
Sonochemical growth of high-density ZnO nanorod arrays	Thin-film	SR = R_g_ − R_a_/R_a_ ca. 8 under 0.1 ppm	250 °C	[44]
Hierarchical ZnO nanostructures by thermal evaporation method	Thick-film	SR = R_g_ − R_a_/R_a_ 0.41 under 1 ppm	200 °C	[45]
ZnO film produced via ion layer adsorption and reaction (SILAR) technique	Thin-film	SR = R_g_ − R_a_/R_a_ 1.37 under 10 ppm	150 °C	[46]
Sheet-like hierarchical ZnO coatings deposited by suspension flame spraying	Thick-film	SR = R_g_ − R_a_/R_a_ 2.6 under 1 ppm	RT + white light	[47]
ZnO nanoparticles produced by separate nucleation and aging steps (SNAS)	Pellet	SR = R_g_/R_a_ ca. 226 under 40 ppm	290 °C	[48]
ZnO submicron rods drop cast on oxidized silicon substrate	Thick-film	SR = R_g_ − R_a_/R_a_ 1 under 1 ppm	RT	[49]
Drop coating of ZnO and Au/ZnO rose-like structures made by microwave-assisted hydrothermal method	n.m.	S = (R_g_ − R_a_)/R_a_ 75 under 5 ppm	300 °C	[50]
ZnO nanoparticles precipitated on sepiolite needles	Thick-film	SR = R_a_/R_g_ ca. 1.08 under 1 ppm	300 °C	[51]
ZnO-based glass ceramic sensor	Thick-film	SR = R_g_/R_a_ ca. 17 under 1 ppm	150 °C	This work

^1^ n.m.: not mentioned.
